# Lactate-to-Hemoglobin Ratio as a Predictor of 30-Day Mortality in Patients with Non-Variceal Acute Upper Gastrointestinal Bleeding

**DOI:** 10.3390/diagnostics16172681

**Published:** 2026-08-22

**Authors:** Muge Gul Gulecoglu Onem, Ali Serel, Mehmet Emin Arayici, İbrahim Ethem Güven, Civanmert Bayrak, Suleyman Dolu, Soner Onem

**Affiliations:** 1 Department of Clinical Biochemistry, Samsun City Hospital, Samsun 55080, Turkey; mugegl@hotmail.com; 2 Department of Internal Medicine, Bozyaka Research and Training Hospital, Izmir 35170, Turkey; ali_serel@hotmail.com; 3 Department of Biostatistics and Medical Informatics, Faculty of Medicine, Dokuz Eylül University, Izmir 35330, Turkey; mehmet.e.arayici@gmail.com; 4 Department of Gastroenterology, Faculty of Medicine, Hacettepe University, Ankara 06230, Turkey; drethemg@gmail.com; 5 Department of Gastroenterology, Faculty of Medicine, Dokuz Eylül University, Izmir 35330, Turkey; bayrakcivanmert@gmail.com (C.B.); soneronem@hotmail.com (S.O.)

**Keywords:** upper gastrointestinal bleeding, mortality, lactate to hemoglobin ratio, risk stratification

## Abstract

**Background/Objectives**: The study aimed to investigate the significance of laboratory biomarkers, particularly the lactate-to-hemoglobin ratio at admission in predicting 30-day mortality in patients with acute upper gastrointestinal bleeding (UGIB). **Methods**: Patients who presented to the emergency department due to acute UGIB between January and December 2024 were included in the study. The laboratory parameters at the time of admission, medication usage, endoscopic diagnoses, and 30-day mortality rates of the patients were investigated. It was investigated whether there were differences in biochemical parameters such as hemoglobin, hematocrit, neutrophil, lymphocyte, platelet, international normalized ratio (INR), pH, lactate, blood urea nitrogen (BUN), creatinine, C-reactive protein (CRP), albumin, BUN to creatinine ratio, and lactate to hemoglobin ratio between patients with and without 30-day mortality. In addition, the relationship between mortality, drug use, and endoscopic diagnoses was examined. **Results**: A total of 241 patients participated in the study. Of the participants in the study, 79 (32.8%) were women and 162 (67.2%) were men. The average age of the patients was 70 years. The most common causes of bleeding were, in order, bulbar ulcer (*n* = 84, 34.9%) and gastric ulcer (*n* = 79, 32.8%). A total of 15 (6.2%) patients developed mortality within 30 days. In the mortality group, lymphocyte (*p* = 0.016) and albumin (*p* = 0.026) values were statistically low, while INR (*p* = 0.041), creatinine (*p* = 0.020), CRP (*p* < 0.01), lactate (*p* = 0.014) and lactate to Hb ratio (LHR) (*p* = 0.012) values were significantly high. In cases of bleeding due to malignancy, mortality was higher. No association was found between medication use and mortality. **Conclusions**: In this exploratory study, the LHR showed potential as an adjunctive biomarker for estimating 30-day mortality in patients with non-variceal acute UGIB. It can be readily calculated based on common laboratory values, does not necessitate complex scoring systems, and can help with quick risk assessment in emergency conditions. Large, multicenter prospective studies are required to confirm prediction accuracy as well as identify optimum cutoff values for clinical application.

## 1. Introduction

Upper gastrointestinal bleeding (UGIB) is a prominent gastrointestinal disorder encountered in emergency clinics [1]. The expected annual incidence of acute upper gastrointestinal bleeding is between 100 and 200 per 100,000 individuals [2]. Despite advancements in treatment, a 5–10% mortality rate can still be observed [3,4]. Quick detection of high-risk patients and the necessity for immediate measures, including drugs, blood transfusion, urgent endoscopic procedures, transcatheter arterial embolization, or surgical intervention, may enhance results. The main risk classification systems for upper gastrointestinal bleeding in daily practice are the Glasgow Blatchford Score (GBS), AIMS65, and the pre-endoscopic Rockall Score.

Clinical signs, including hypotension, postural hypotension, and tachycardia, support primary and specialist physicians in differentiating between severe and non-severe UGIB.

Nevertheless, these clinical findings are not consistently accurate, particularly in elderly individuals who have many comorbidities and those on multiple drugs that might affect vital signs. Furthermore, the initial hemoglobin and hematocrit levels may not accurately represent a patient’s true intravascular volume status, as they can be erroneously raised due to hemoconcentration or influenced by a persistent anemic condition alongside the acute bleeding episode [5,6].

A number of variables, including age, albumin levels, consciousness, heart rate, systolic blood pressure, and international normalized ratio (INR), can be utilized for estimating mortality risk prior to endoscopy [7,8]. The lactate/hemoglobin ratio (LHR) has been found to be effective in predicting mortality [9]. Studies have been conducted to investigate the blood urea nitrogen (BUN)/creatinine ratio in differentiating between upper and lower gastrointestinal bleeding [10]. Additionally, it has been found to be related to the need for transfusion requirement in determining the severity of bleeding [11,12]. To our knowledge, data regarding LHR in UGIB remain limited.

The primary endpoint of this study is to investigate the role of complete blood count and biochemical parameter pH, lactate, BUN, creatinine, albumin, C-reactive protein (CRP), LHR, and BUN/creatinine in predicting 30-day mortality. The secondary endpoint is to investigate whether endoscopic diagnosis and drug use are associated with mortality. Given the limited available evidence regarding LHR in non-variceal UGIB, this study was designed as an exploratory assessment rather than a definitive validation of its prognostic performance.

## 2. Materials and Methods

This study was performed as a retrospective cohort study. The patients diagnosed with UGIB who were admitted to the Samsun Research and Training Hospital via the emergency department (ED) between January and December 2024 enrolled for the study. The demographic characteristics of the patients, endoscopic findings, laboratory parameters, and whether there was mortality within 30 days were investigated.

Laboratory parameters such as hemoglobin, hematocrit, neutrophil, lymphocyte, platelet, INR, pH, lactate, BUN, creatinine, CRP, albumin, BUN/creatinine, and lactate/hemoglobin values at admission were measured. Serum C-reactive protein (CRP) levels were measured using the routine quantitative assay in the central biochemistry laboratory; high-sensitivity CRP (hs-CRP) was not used.

Patients underwent gastroscopy within the first 24 h after admission after adequate resuscitation. Endoscopic diagnoses were recorded. Patients were divided into two groups: those with and without 30-day mortality. All-cause 30-day mortality was obtained from the hospital data system and the national health information system. The difference between those who died within 30 days and those who did not, based on laboratory parameters at the time of application, was investigated.

Patients under 18 years old, with insufficient data, with bleeding due to esophageal varices, and who experienced bleeding during hospitalization were excluded from the study.

The investigation was executed after the Samsun Education and Research Hospital Clinical Research Ethics Committee granted approval for the procedure numbered 2026/1/11 on 25 February 2026. This study complies with the principles of the Helsinki Declaration.

### Statistical Analyses

Statistical analyses were performed to evaluate clinical, laboratory, endoscopic, and treatment-related factors associated with 30-day mortality. Patients were divided into two groups according to mortality status: survivors and non-survivors. The primary dependent variable was mortality, which was coded as a binary outcome. The distribution of continuous variables was assessed visually and analytically. Since most continuous variables did not show a normal distribution and the number of patients in the mortality group was limited, continuous variables were summarized as median and interquartile range (IQR). Categorical variables were presented as number and percentage. Comparisons between survivors and non-survivors were performed using the Mann–Whitney U test for continuous variables. Categorical variables were compared using the chi-square test when the assumptions were met. When expected cell counts were small, the Fisher’s exact test or Fisher–Freeman–Halton exact test was used, as appropriate. For the evaluation of predictors of mortality, binary logistic regression analysis was performed. First, univariable logistic regression analyses were conducted for all clinically and statistically appropriate variables. Results were reported as odds ratios (ORs) with 95% confidence intervals (CIs) and *p* values. Variables that were statistically significant in the univariable analysis were considered candidates for the multivariable logistic regression model. Because only 15 mortality events occurred, the multivariable logistic regression analysis was considered exploratory and was performed primarily to assess potential independent associations rather than to develop a definitive prediction model. However, because the number of mortality events was limited, the number of variables included in the multivariable model was restricted to reduce the risk of model overfitting. In addition, variables with potential collinearity were not included in the same multivariable model. For clinically interpretable risk stratification, selected continuous variables were also categorized according to predefined cut-off values. Receiver operating characteristic (ROC) curve analysis was performed to determine the optimal cut-off values of selected biomarkers for predicting mortality. The optimal thresholds were identified using Youden’s index, and the corresponding cut-off values were used to categorize lactate/hemoglobin ratio, lactate, and CRP for subsequent logistic regression analyses. The following thresholds were used: lactate/hemoglobin ratio ≥ 0.26, lactate ≥ 2.55 mmol/L, and CRP ≥ 8.8 mg/L. Lactate was evaluated in the univariable analysis but was excluded from the multivariable model because of its collinearity with the lactate/hemoglobin ratio. Model performance was assessed using discrimination and calibration measures. Discriminative ability was evaluated using the area under the receiver operating characteristic curve (AUC). Calibration of the multivariable models was assessed using the Hosmer–Lemeshow goodness-of-fit test. A non-significant Hosmer–Lemeshow test result was interpreted as indicating no evidence of poor model fit. All statistical analyses were performed using IBM SPSS Statistics for Mac, version 30.0 (IBM Corp., Armonk, NY, USA) and STATA Statistical Software, release 18 (StataCorp LLC, College Station, TX, USA). Statistical significance was defined as a two-tailed *p* value < 0.05.

## 3. Results

A total of 241 patients were included in the study. Of these, 226 patients were classified as survivors and 15 patients as non-survivors. The median age of the overall cohort was 70 years (IQR, 58–79). Median age was 70 years (IQR, 57–79) among survivors and 75 years (IQR, 63–80) among non-survivors, with a *p* value of 0.468. Overall, 162 patients were male (67.2%) and 79 were female (32.8%). Among survivors, 150 patients were male (66.4%) and 76 were female (33.6%). Among non-survivors, 12 patients were male (80.0%) and 3 were female (20.0%). The *p* value for sex distribution was 0.397. Regarding medication use, 76 patients had no recorded medication use or were classified as none/not recorded (31.5%).

The most frequent medication categories were NSAID use in 35 patients (14.5%), ASA use in 32 patients (13.3%), DOAC use in 32 patients (13.3%), and combination therapy in 25 patients (10.4%). The *p* value for medication use across mortality groups was 0.248. The most common gastroscopic findings in the overall cohort were bulbar ulcer in 84 patients (34.9%) and gastric ulcer in 79 patients (32.8%). Malignant lesions were detected in 21 patients (8.7%), including 16 survivors (7.1%) and 5 non-survivors (33.3%). The *p* value for gastroscopic findings across mortality groups was 0.025. Baseline demographic, clinical, medication-related, and endoscopic characteristics of the study population are presented in Table 1.

Baseline laboratory characteristics of the study population are presented in Table 2. In the overall cohort, the median WBC count was 10.19 × 10^3^/µL (IQR, 7.90–13.10), the median hemoglobin level was 8.4 g/dL (IQR, 7.0–10.9), and the median hematocrit was 26.0% (IQR, 22.0–33.0). The median lymphocyte count was 1.90 × 10^3^/µL (IQR, 1.31–2.80) in survivors and 1.04 × 10^3^/µL (IQR, 0.89–2.38) in non-survivors (*p* = 0.016). The median INR was 1.07 (IQR, 0.99–1.18) among survivors and 1.17 (IQR, 1.10–1.48) among non-survivors (*p* = 0.041). The median lactate level was 1.8 mmol/L (IQR, 1.2–2.5) in survivors and 3.0 mmol/L (IQR, 1.5–5.2) in non-survivors (*p* = 0.014). The median creatinine level was 0.99 mg/dL (IQR, 0.80–1.34) in survivors and 1.53 mg/dL (IQR, 0.91–2.07) in non-survivors (*p* = 0.020). The median CRP level was 5.0 mg/L (IQR, 2.0–22.0) among survivors and 43.0 mg/L (IQR, 18.0–53.5) among non-survivors (*p* < 0.001). The median albumin level was 3.3 g/dL (IQR, 3.0–3.7) in survivors and 3.0 g/dL (IQR, 2.7–3.4) in non-survivors (*p* = 0.026). The median lactate/hemoglobin ratio was 0.19 (IQR, 0.13–0.33) in survivors and 0.39 (IQR, 0.22–0.73) in non-survivors (*p* = 0.012). No statistically significant differences were observed between survivors and non-survivors in WBC count, hemoglobin, hematocrit, platelet count, pH, BUN, or BUN/creatinine ratio.

**Figure 1:**
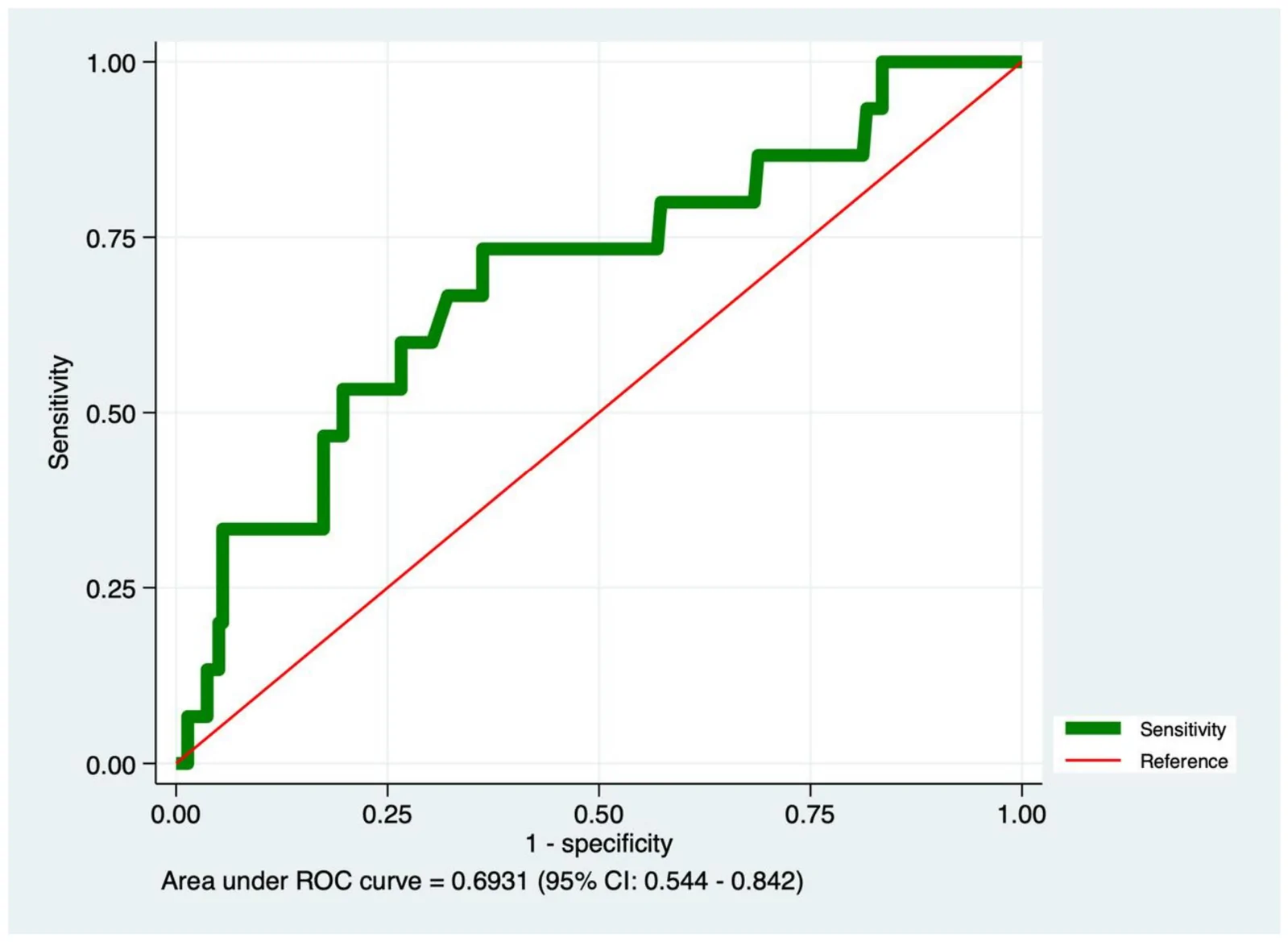
ROC curve analysis for lactate-to-hemoglobin ratio.

**Figure 2:**
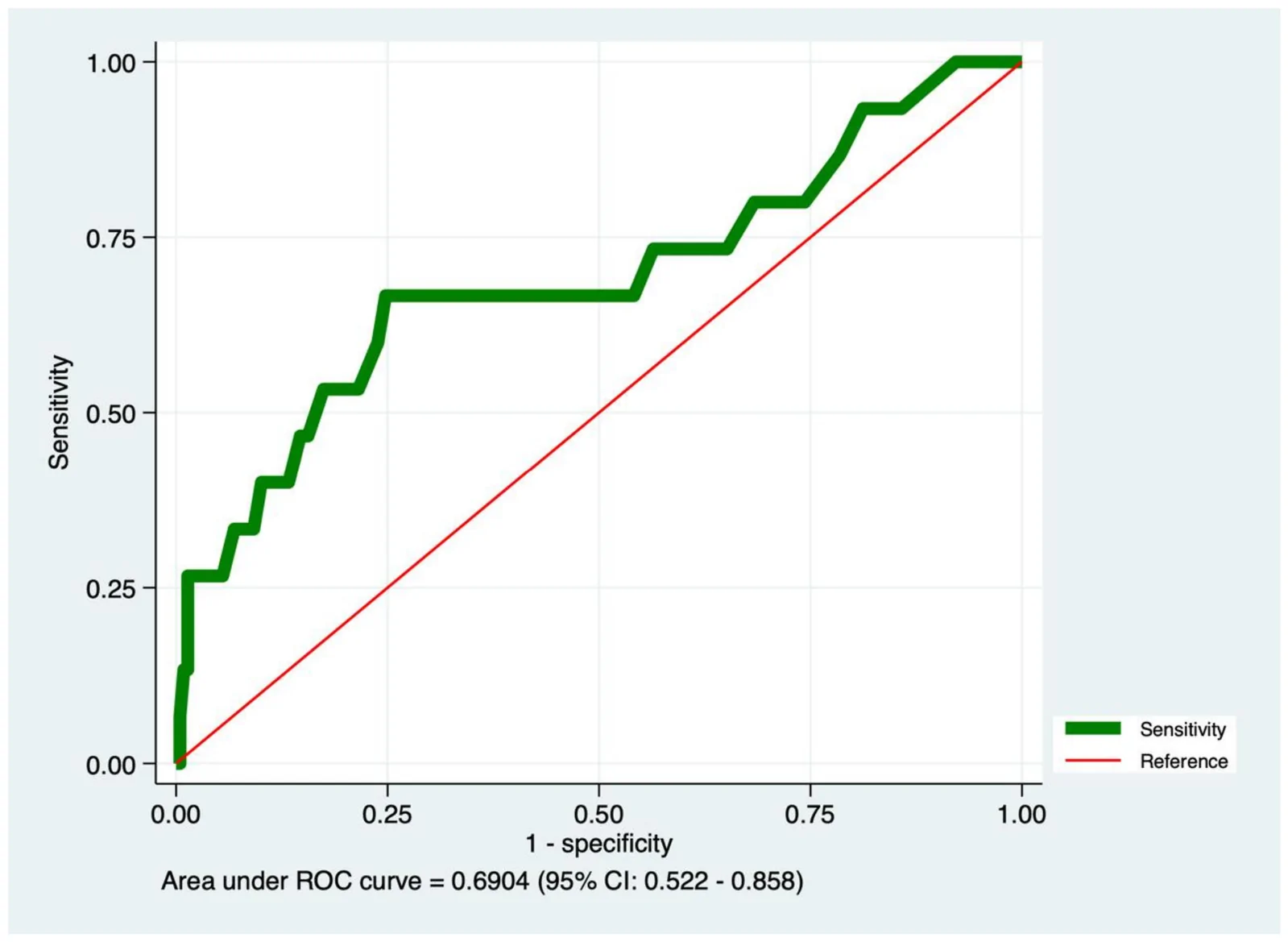
ROC curve analysis for lactate.

**Figure 3:**
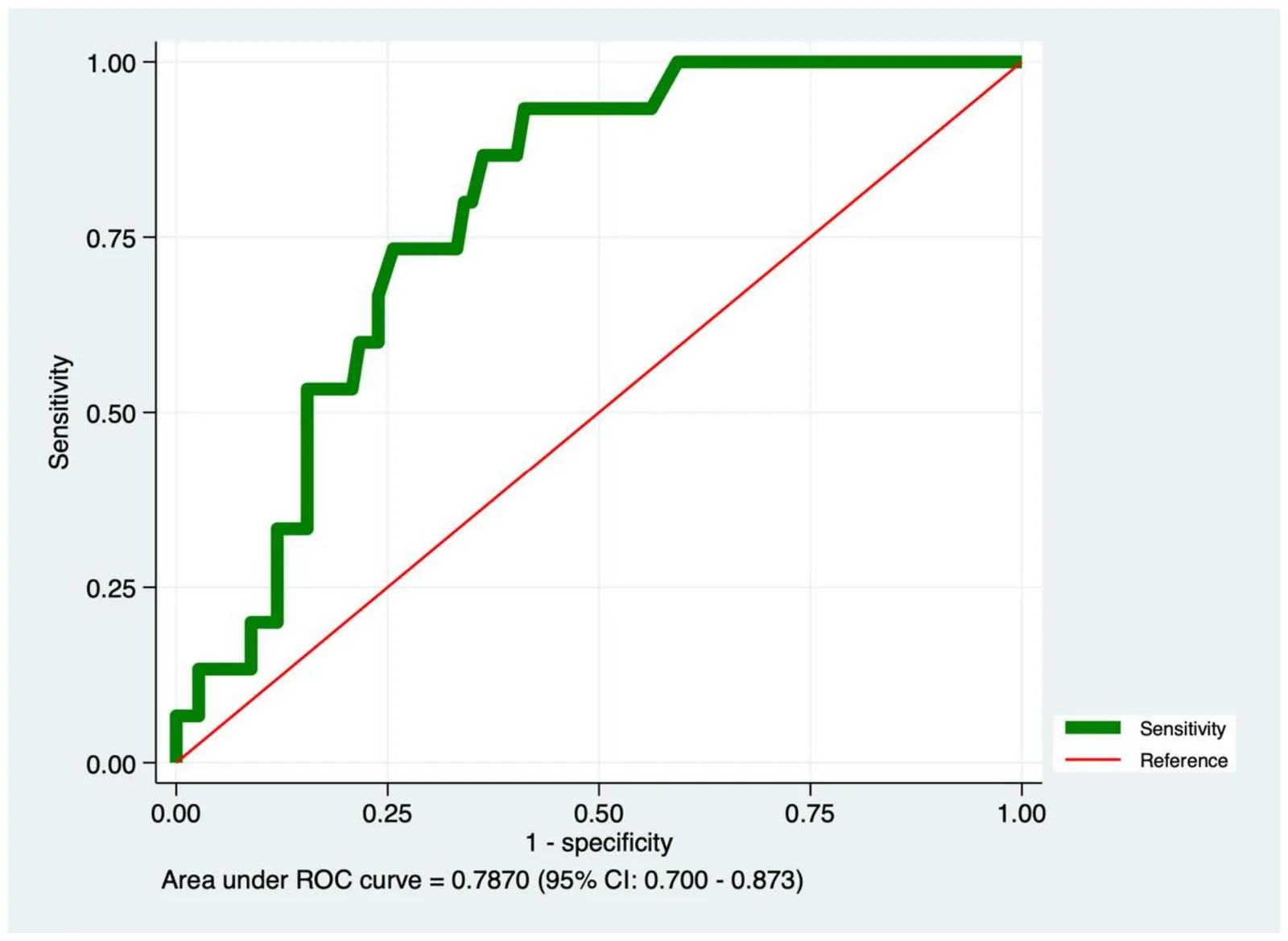
ROC curve analysis for C-reactive protein.

ROC curve analysis was performed to evaluate the predictive performance of lactate/Hb ratio, lactate, and CRP for 30-day mortality. The optimal cut-off value for the lactate/Hb ratio was 0.26 (Figure 1), with a sensitivity of 73.33%, specificity of 63.76%, and AUC of 0.693 (95% CI, 0.544–0.842; *p* = 0.009). For lactate, the optimal cut-off value was 2.55 mmol/L (Figure 2), with a sensitivity of 66.6%, specificity of 75.23%, and AUC of 0.690 (95% CI, 0.522–0.858; *p* = 0.022). For CRP, the optimal cut-off value was 8.8 mg/L (Figure 3), with a sensitivity of 93.3%, specificity of 58.8%, and AUC of 0.787 (95% CI, 0.700–0.873; *p* < 0.001). The specific ROC analysis results are presented in Table 3.

Univariable logistic regression analysis for mortality was undertaken and is presented in Table 4. In the univariable analysis, malignant lesion was associated with mortality (OR, 6.56; 95% CI, 2.00–21.52; *p* = 0.002). Albumin was also associated with mortality, with an OR of 0.24 per 1 g/dL increase (95% CI, 0.08–0.69; *p* = 0.008). Among the cut-off-based biochemical variables, a lactate/Hb ratio ≥ 0.26 was associated with mortality (OR, 4.84; 95% CI, 1.49–15.70; *p* = 0.009). Lactate ≥ 2.55 mmol/L was associated with mortality (OR, 6.07; 95% CI, 1.99–18.55; *p* = 0.002). CRP ≥ 8.8 mg/L was also associated with mortality (OR, 20.02; 95% CI, 2.59–154.90; *p* = 0.004). Age, sex, any medication use, pH, WBC, hemoglobin, hematocrit, neutrophil count, lymphocyte count, platelet count, INR, BUN, and creatinine were not statistically significant in the univariable logistic regression analysis.

The multivariable logistic regression model for mortality is presented in Table 5. In this model, a lactate/hemoglobin ratio ≥ 0.26 was associated with mortality after adjustment (adjusted OR, 4.88; 95% CI, 1.36–17.44; *p* = 0.015). CRP ≥ 8.8 mg/L was also associated with mortality (adjusted OR, 14.83; 95% CI, 1.86–117.98; *p* = 0.011). Malignant lesion remained associated with mortality in the adjusted model (adjusted OR, 5.19; 95% CI, 1.34–20.08; *p* = 0.017). The primary model included 233 patients and 15 mortality events. The AUC of the model was 0.857, and the AIC was 90.05. The Hosmer–Lemeshow goodness-of-fit test yielded χ^2^ = 3.80 with 6 degrees of freedom and a *p* value of 0.704.

## 4. Discussion

Acute upper gastrointestinal bleeding is a condition that carries a risk of mortality despite medical, endoscopic, interventional, and surgical treatments. In a multicenter study involving 1020 patients, the 30-day mortality rate was determined to be 4.5% [13]. In our series, mortality was observed in 6.2% of a total of 15 patients. It was similar to the rates reported in the literature. Simple, fast, and easily accessible parameters or scoring systems at the time of application can help identify high-risk patient groups. Early identification of high-risk patients is essential for timely resuscitation, intensive monitoring, and appropriate therapeutic interventions. In the present study, we investigated the prognostic value of several readily available laboratory parameters in patients presenting with UGIB and demonstrated that the lactate-to-hemoglobin ratio (LHR) was independently associated with 30-day mortality. In addition, elevated lactate, creatinine, and CRP levels, as well as low albumin and lymphocyte counts, were associated with mortality in univariable analyses. Furthermore, the detection of a malignant lesion during gastroscopy has been identified as a risk factor for 30-day mortality. At the time of admission to the emergency department, these parameters can be quickly evaluated and may help identify patients at increased risk who could benefit from closer monitoring and timely clinical management.

In a study involving 589 patients, the average age was 67 years. The proportion of men was 66.2% [14]. In another series that included 311 patients, the average age was 70 years, and 65% of the patients were male [15]. Upper gastrointestinal bleeding is more common in male patients. Studies have found male-to-female ratios ranging from 1.7 to 1.8 [2]. In our study, consistent with the literature, there were more male patients, with a male/female ratio of 2 and an average age of 70 years. The effect of age and gender on mortality was not identified.

Peptic ulcer diseases remained the leading cause of acute UGIB, accounting for up to 67% of all cases [16]. There are also publications indicating that it is observed between 35–50% [17]. The most common causes of acute upper gastrointestinal bleeding in our series were, in order, duodenal and gastric ulcers. This finding is consistent with previous epidemiological studies reporting peptic ulcer disease as the leading cause of non-variceal UGIB. When examining the relationship between etiology and 30-day mortality, it was found that mortality was higher in patients with malignant lesions. Mortality developed in 5 out of 21 patients who were followed for upper gastrointestinal mass bleeding. Alternative treatment methods should be considered for patients presenting with massive bleeding due to the limitations of endoscopic treatments.

In a study of 131 patients with upper gastrointestinal bleeding, the Hb level at admission was found to be 8.2 g/dL [18]. In the study conducted by Güner and colleagues, the hemoglobin level was found to be 9 g/dL, while the hematocrit level was 27.8% [14]. In our patient group, the admission values were found to be consistent with the literature. However, no correlation was found between hemoglobin and hematocrit values in predicting mortality.

Antiplatelet, anticoagulant, or NSAID drug use are medications that increase the risk of gastrointestinal bleeding. In the series involving geriatric patients, no relationship was found between antiplatelet, anticoagulant, NSAI drugs and mortality [19]. The most frequently used medications in our series were NSAIDs (14.5%) and ASA (13.3%). No association was found between mortality and the use of medications.

Lactate is the end product of anaerobic glycolysis. The increase in lactate production is usually caused by impaired tissue oxygenation, resulting from either reduced oxygen delivery or a defect in mitochondrial oxygen utilization. In patients presenting with upper gastrointestinal bleeding, tissue hypoxia that begins after a decrease in circulating blood volume also increases lactate levels [20]. In another study examining lactate and mortality in lower and upper gastrointestinal bleeding, it was found that a lactate level above 4 mmol/L increased the risk of mortality by 6 times [21]. In our study, the average lactate level in the mortality group was 3.0 mmol/L, which was significantly higher than in the other group. The cutoff point was found to be 2.55 mmol/L in our study. In another recent study, it was stated that the lactate/hemoglobin ratio, by combining these two parameters, provides a more comprehensive assessment that integrates both the severity of blood loss and the resulting systemic hypoxia. Thus, it may provide an adjunctive prognostic marker. The lactate/hemoglobin ratio was found to be associated with mortality in 1387 patients [9]. In our study, the lactate/hemoglobin ratio was also found to be effective in predicting mortality.

BUN is a measure of the amount of urea nitrogen in the blood [22]. BUN represents the terminal products of protein metabolism via ammonia. When upper GI bleeding occurs, the blood is digested to protein. This protein is transported to the liver via the portal vein and metabolized to BUN in the urea cycle. Higher BUN values are therefore associated with the digestion of blood [23]. Patients with a BUN/creatinine ratio > 23.3 had significantly higher rates of red blood cell transfusion, endoscopic intervention, and mortality compared to those with a BUN/creatinine ratio ≤ 23.3 [24]. In a different study, by Wu et al., the BUN/creatinine ratio over 30 was discovered as being an independent risk factor for mortality in patients with upper GI bleeding [25]. Our study did not find any relationship between the BUN/creatinine ratio and mortality.

An elevated INR, indicative of coagulopathy, is frequently observed in GIB patients and is significantly correlated with more severe hemorrhage and increased mortality [26]. In a 2025 study involving 1557 patients, the INR level was found to be significantly higher in the mortality group (1.4 vs. 1.7) [27]. In our series, the average INR level was higher in the mortality group.

Inflammatory stimulants (infection, trauma, ischemia, burns, etc.) result in the release of cytokines (IL-1β, IL-6, IL-8, TNF-α, interferon-γ, transforming growth factor-β, etc.) from macrophages and monocytes at the site of inflammation. These cytokines cause an increase in the production and release of acute phase proteins, such as CRP, ferritin, and fibrinogen, and a concomitant decrease in the release of negative acute phase reactants, such as albumin. Studies have shown that high CRP and low albumin levels are associated with mortality in gastrointestinal bleeding [28]. In a study involving 596 patients, the mortality rate was found to be 6%, and the CRP value (23 vs. 41 mg/L) was higher in the mortality group [29]. Elevated WBC is associated with the severity and mortality rate of upper GI bleeding [30]. In our study, CRP was found to be significantly higher in the mortality group, while albumin levels were significantly lower. The cutoff point for CRP in our study was 8.8 mg/L.

The limitations of this study include its retrospective single-center design and the relatively small number of patients who died within 30 days. The limited number of mortality events may have compromised the stability of the multivariable analysis, resulting in relatively wide confidence intervals. Accordingly, the multivariable findings should be interpreted as exploratory estimates rather than definitive measures of independent prognostic effect. In addition, established UGIB risk scores, such as the Glasgow-Blatchford Score and Rockall Score, were not evaluated for comparison. Finally, the potential impact of underlying comorbidities and malignancies on all-cause 30-day mortality was not specifically investigated. In addition, all laboratory measurements were performed in a single center using the same analyzers and standardized laboratory protocols. Therefore, differences in assay methods, analytical platforms, and commercial reagents across institutions may influence laboratory values and the proposed cutoff values, which may limit the external generalizability of our findings.

Our findings should be interpreted as an exploratory evaluation of LHR in a narrowly defined cohort of patients with non-variceal acute UGIB rather than as evidence establishing a novel prognostic biomarker. While LHR demonstrated an independent association with mortality, the current results primarily support further investigation and external validation rather than immediate clinical implementation.

Although the lactate-to-hemoglobin ratio demonstrated a statistically significant prognostic value, its discriminative performance was moderate (AUC = 0.693). Therefore, it should be regarded as an adjunctive marker to support, rather than replace, existing clinical assessment and risk stratification strategies.

Early stratification of bleeding severity will increasingly become a key element in the management of GIB patients. An ideal risk score should be simple, straightforward to use, and highly effective. Our study showed that mortality was associated with parameters such as lactate, lactate/Hb ratio, CRP, and albumin, which are easily and quickly accessible in emergency departments.

In conclusion, this exploratory study suggests that the lactate-to-hemoglobin ratio may have potential as an adjunctive biomarker for early mortality risk assessment in patients with non-variceal acute upper gastrointestinal bleeding. Although an elevated lactate-to-hemoglobin ratio was independently associated with 30-day mortality, the exploratory nature of the study and the limited number of mortality events warrant a cautious interpretation of these findings. Larger prospective multicenter studies are needed to validate these results, determine optimal cutoff values, and compare the predictive performance of the lactate-to-hemoglobin ratio with established UGIB risk scoring systems. Future studies may also explore whether incorporating LHR into existing risk models could further improve prognostic performance.

## Figures and Tables

**Table 1:** Baseline demographic, clinical, and endoscopic characteristics of the study population.

Characteristic	All Patients (*N* = 241)	Survivors (*n* = 226)	Non-Survivors (*n* = 15)	*p* Value
Age, years	70 (58–79)	70 (57–79)	75 (63–80)	0.468
**Sex, *n* (%)**				0.397
Male	162 (67.2)	150 (66.4)	12 (80.0)	
Female	79 (32.8)	76 (33.6)	3 (20.0)	
**Medication use, *n* (%)**				0.248
None/not recorded	76 (31.5)	72 (31.9)	4 (26.7)	
NSAID	35 (14.5)	35 (15.5)	0 (0.0)	
ASA	32 (13.3)	30 (13.3)	2 (13.3)	
Warfarin	15 (6.2)	14 (6.2)	1 (6.7)	
Clopidogrel	22 (9.1)	19 (8.4)	3 (20.0)	
DOAC	32 (13.3)	30 (13.3)	2 (13.3)	
Combination therapy	25 (10.4)	23 (10.2)	2 (13.3)	
LMWH	4 (1.7)	3 (1.3)	1 (6.7)	
**Gastroscopy finding, *n* (%)**				**0.025** †
Bulbar ulcer	84 (34.9)	78 (34.5)	6 (40.0)	
Gastric ulcer	79 (32.8)	78 (34.5)	1 (6.7)	
Esophagitis	12 (5)	11 (4.8)	1 (6.7)	
Mallory–Weiss lesion	14 (5.8)	14 (6.2)	0 (0.0)	
Malignant lesion	21 (8.7)	16 (7.1)	5 (33.3)	
Anastomotic ulcer	14 (5.8)	13 (5.8)	1 (6.7)	
Angiodysplasia	11 (4.6)	11 (4.9)	0 (0.0)	
Dieulafoy lesion	5 (2.1)	4 (1.8)	1 (6.7)	
Polyp	1 (0.4)	1 (0.4)	0 (0.0)	

ASA, acetylsalicylic acid; DOAC, direct oral anticoagulant; LMWH, low-molecular-weight heparin; NSAID, non-steroidal anti-inflammatory drug. Continuous variables were compared using the Mann–Whitney U test. Categorical variables were compared using Fisher’s exact test for 2 × 2 tables and the Fisher–Freeman–Halton exact test with Monte Carlo simulation for larger tables. Percentages were calculated using available non-missing data. † Statistically significant.

**Table 2:** Baseline laboratory characteristics of the study population.

Characteristic	All Patients (*N* = 241)	Survivors (*n* = 226)	Non-Survivors (*n* = 15)	*p* Value
WBC (×10^3^/µL)	10.19 (7.90–13.10)	10.18 (7.83–12.88)	12.50 (8.15–16.00)	0.173
Hemoglobin (g/dL)	8.4 (7.0–10.9)	8.5 (7.0–11.0)	8.0 (7.3–8.7)	0.283
Hematocrit (%)	26.0 (22.0–33.0)	26.0 (21.8–33.8)	24.0 (23.0–28.5)	0.491
Neutrophil (×10^3^/µL)	6.97 (5.30–9.93)	6.90 (5.22–9.86)	9.02 (6.79–11.54)	0.078
Lymphocyte (×10^3^/µL)	1.84 (1.26–2.80)	1.90 (1.31–2.80)	1.04 (0.89–2.38)	**0.016 †**
Platelet (×10^3^/µL)	253 (188–326)	253 (189–324)	208 (148–386)	0.823
INR	1.08 (0.99–1.20)	1.07 (0.99–1.18)	1.17 (1.10–1.48)	**0.041 †**
pH	7.36 (7.32–7.40)	7.36 (7.33–7.40)	7.35 (7.28–7.41)	0.296
Lactate (mmol/L)	1.9 (1.2–2.7)	1.8 (1.2–2.5)	3.0 (1.5–5.2)	**0.014 †**
BUN (mg/dL)	34.6 (23.4–53.2)	33.9 (21.6–53.2)	40.2 (30.6–50.9)	0.252
Creatinine (mg/dL)	1.00 (0.81–1.39)	0.99 (0.80–1.34)	1.53 (0.91–2.07)	**0.020 †**
BUN/Cr ratio	32.6 (20.9–43.8)	32.7 (21.1–43.8)	29.9 (19.8–37.2)	0.349
CRP (mg/L)	5.9 (2.0–28.0)	5.0 (2.0–22.0)	43.0 (18.0–53.5)	**<0.001 †**
Albumin (g/dL)	3.3 (3.0–3.7)	3.3 (3.0–3.7)	3.0 (2.7–3.4)	**0.026 †**
Lactate/hemoglobin ratio	0.20 (0.13–0.35)	0.19 (0.13–0.33)	0.39 (0.22–0.73)	**0.012 †**

BUN, blood urea nitrogen; CRP, C-reactive protein; INR, international normalized ratio; WBC, white blood cell count. Continuous variables were compared using the Mann–Whitney U test. Percentages were calculated using available non-missing data. Missing data: pH, *n* = 9; lactate, *n* = 8; INR, *n* = 7; creatinine, *n* = 1; albumin, *n* = 26; lactate/hemoglobin ratio, *n* = 8. † Statistically significant values are shown in bold.

**Table 3:** Cut-off value, sensitivity, and specificity of the main biochemical variables in the prediction of 30-day mortality.

	Cut-Off Value	Sensitivity (%)	Specificity (%)	AUC	95% CI for AUC	*p*-Value
Lactate/Hb	0.26	73.33%	63.76%	0.693	0.544–0.842	**0.009 †**
Lactate	2.55	66.6%	75.23%	0.690	0.522–0.858	**0.022 †**
CRP	8.8	93.3%	58.8%	0.787	0.700–0.873	**<0.001 †**

AUC: area under curve; CRP: C-reactive protein; Hb: hemoglobin. CI: confidence interval. † Statistically significant values are shown in bold.

**Table 4:** Univariable logistic regression analysis for mortality.

Variable	Unit/Comparison	*n*/Events	Univariable OR (95% CI)	*p* Value
Age	per 10-year increase	241/15	1.18 (0.81–1.71)	0.399
Male sex	male vs. female	241/15	2.03 (0.56–7.40)	0.285
Any medication use	yes vs. none/not recorded	241/15	1.29 (0.40–4.18)	0.676
Malignant lesion on gastroscopy	yes vs. other findings	241/15	6.56 (2.00–21.52)	**0.002 †**
pH	per 0.1-unit increase	232/15	0.59 (0.29–1.22)	0.158
WBC	per 1 × 10^3^/µL increase	241/15	1.09 (0.97–1.22)	0.157
Hemoglobin	per 1 g/dL increase	241/15	0.88 (0.72–1.09)	0.236
Hematocrit	per 1% increase	241/15	0.97 (0.91–1.04)	0.388
Neutrophil	per 1 × 10^3^/µL increase	241/15	1.11 (0.97–1.26)	0.128
Lymphocyte	per 1 × 10^3^/µL increase	241/15	0.53 (0.28–1.01)	0.052
Platelet	per 100 × 10^3^/µL increase	241/15	1.19 (0.77–1.84)	0.427
INR	per 0.1-unit increase	234/14	1.02 (1.00–1.04)	0.050
BUN	per 10 mg/dL increase	241/15	1.02 (0.93–1.12)	0.655
Creatinine	per 1 mg/dL increase	240/15	1.27 (0.96–1.67)	0.089
Albumin	per 1 g/dL increase	215/13	0.24 (0.08–0.69)	**0.008 †**
Lactate/Hb ≥ 0.26	≥0.26 vs. <0.26	233/15	4.84 (1.49–15.70)	**0.009 †**
Lactate ≥ 2.55 mmol/L	≥2.55 vs. <2.55 mmol/L	233/15	6.07 (1.99–18.55)	**0.002 †**
CRP ≥ 8.8 mg/L	≥8.8 vs. <8.8 mg/L	241/15	20.02 (2.59–154.90)	**0.004 †**

Univariable candidate variables with *p* < 0.05 included gastroscopic malignant lesion, lactate, lactate ≥ 2.55 mmol/L, CRP, CRP ≥ 8.8 mg/L, albumin, lactate/hemoglobin ratio, and lactate/hemoglobin ratio ≥ 0.26. ORs are expressed per the unit/comparison shown. † Statistically significant values are shown in bold.

**Table 5:** Multivariable logistic regression model for mortality.

Variable	Adjusted OR	95% Cl	*p* Value
Lactate/hemoglobin ratio ≥ 0.26	4.88	1.36–17.44	**0.015 †**
CRP ≥ 8.8 mg/L	14.83	1.86–117.98	**0.011 †**
Gastroscopic malignant lesion	5.19	1.34–20.08	**0.017 †**

Model performance: *N* = 233, mortality events = 15, AUC = 0.857, AIC = 90.05. Hosmer–Lemeshow χ^2^ = 3.80, df = 6, *p* = 0.704. † Statistically significant values are shown in bold.

## Data Availability

The original contributions presented in this study are included in the article. Further inquiries can be directed to the corresponding author.

## References

[1] Peery A.F., Crockett S.D., Barritt A.S., Dellon E.S., Eluri S., Gangarosa L.M., Jensen E.T., Lund J.L., Pasricha S., Runge T. (2015). Burden of Gastrointestinal, Liver, and Pancreatic Diseases in the United States. Gastroenterology.

[2] Rockall T.A., Logan R.F., Devlin H.B., Northfield T.C. (1995). Incidence of and mortality from acute upper gastrointestinal haemorrhage in the United Kingdom. Steering Committee and members of the National Audit of Acute Upper Gastrointestinal Haemorrhage. BMJ.

[3] Barkun A., Sabbah S., Enns R., Armstrong D., Gregor J., Fedorak R.N., Rahme E., Toubouti Y., Martel M., Chiba N. (2004). The Canadian Registry on Nonvariceal Upper Gastrointestinal Bleeding and Endoscopy (RUGBE): Endoscopic hemostasis and proton pump inhibition are associated with improved outcomes in a real-life setting. Am. J. Gastroenterol..

[4] Laine L., Yang H., Chang S.C., Datto C. (2012). Trends for incidence of hospitalization and death due to GI complications in the United States from 2001 to 2009. Am. J. Gastroenterol..

[5] Wiesen A.R., Hospenthal D.R., Byrd J.C., Glass K.L., Howard R.S., Diehl L.F. (1994). Equilibration of hemoglobin concentration after transfusion in medical inpatients not actively bleeding. Ann. Intern. Med..

[6] Elizalde J.I., Clemente J., Marín J.L., Panés J., Aragón B., Mas A., Piqué J.M., Terés J. (1997). Early changes in hemoglobin and hematocrit levels after packed red cell transfusion in patients with acute anemia. Transfusion.

[7] Saltzman J.R., Tabak Y.P., Hyett B.H., Sun X., Travis A.C., Johannes R.S. (2011). A simple risk score accurately predicts in-hospital mortality, length of stay, and cost in acute upper GI bleeding. Gastrointest. Endosc..

[8] Hyett B.H., Abougergi M.S., Charpentier J.P., Kumar N.L., Brozovic S., Claggett B.L., Travis A.C., Saltzman J.R. (2013). The AIMS65 score compared with the Glasgow-Blatchford score in predicting outcomes in upper GI bleeding. Gastrointest. Endosc..

[9] Wu Q., Cen F., Xie Y., Ning X., Wang J., Lin Z. (2025). Lactate to hemoglobin ratio predicts short and long term mortality in critically ill patients with Gastrointestinal bleeding. Sci. Rep..

[10] Ernst A.A., Haynes M.L., Nick T.G., Weiss S.J. (1999). Usefulness of the blood urea nitrogen/creatinine ratio in gastrointestinal bleeding. Am. J. Emerg. Med..

[11] Chalasani N., Clark W.S., Wilcox C.M. (1997). Blood urea nitrogen to creatinine concentration in gastrointestinal bleeding: A reappraisal. Am. J. Gastroenterol..

[12] Wang J., Ren H., Gu B., Zhang Y. (2024). Differential Analysis of Blood Routine Examination Parameters in Patients with Upper Gastrointestinal Bleeding and Lower Gastrointestinal Bleeding. Altern. Ther. Health Med..

[13] Marmo R., Koch M., Cipolletta L., Capurso L., Pera A., Bianco M.A., Rocca R., Dezi A., Fasoli R., Rotondano G. (2008). Predictive factors of mortality from nonvariceal upper gastrointestinal hemorrhage: A multicenter study. Am. J. Gastroenterol..

[14] Güner N.G., Çatal F., Yürümez Y., Güneysu F., Bostancı F. (2025). Comparison of the new risk score (ABL) with the Glasgow Blatchford Score, AIMS65, and pre-endoscopic Rockall Score in patients with upper gastrointestinal bleeding admitted to the emergency department. BMC Emerg. Med..

[15] Arıkoğlu S., Tezel O., Büyükturan G., Başgöz B.B. (2025). The efficacy and comparison of upper gastrointestinal bleeding risk scoring systems on predicting clinical outcomes among emergency unit patients. BMC Gastroenterol..

[16] Abraham N.S., Barkun A.N., Sauer B.G., Douketis J., Laine L., Noseworthy P.A., Telford J.J., Leontiadis G.I. (2022). American College of Gastroenterology-Canadian Association of Gastroenterology Clinical Practice Guideline: Management of Anticoagulants and Antiplatelets During Acute Gastrointestinal Bleeding and the Periendoscopic Period. Am. J. Gastroenterol..

[17] Martino A., Di Serafino M., Orsini L., Giurazza F., Fiorentino R., Crolla E., Campione S., Molino C., Romano L., Lombardi G. (2023). Rare causes of acute non-variceal upper gastrointestinal bleeding: A comprehensive review. World J. Gastroenterol..

[18] Fukuda S., Shimodaira Y., Watanabe K., Takahashi S., Sugawara K., Suzuki Y., Watanabe N., Koizumi S., Matsuhashi T., Iijima K. (2020). Risks for Rebleeding and In-Hospital Mortality after Gastrointestinal Bleeding in a Tertiary Referral Center in Japan. Digestion.

[19] Onem S., Güven İ.E., Dolu S., Onem M.G.G., Akyol T. (2025). Effects of antiplatelet, anticoagulant, and nonsteroidal anti-inflammatory drugs on upper gastrointestinal bleeding in geriatric patients. Medicine.

[20] Wada T., Hagiwara A., Uemura T., Yahagi N., Kimura A. (2016). Early lactate clearance for predicting active bleeding in critically ill patients with acute upper gastrointestinal bleeding: A retrospective study. Intern. Emerg. Med..

[21] Shah A., Chisolm-Straker M., Alexander A., Rattu M., Dikdan S., Manini A.F. (2014). Prognostic use of lactate to predict inpatient mortality in acute gastrointestinal hemorrhage. Am. J. Emerg. Med..

[22] Schutz Y. (2011). Protein turnover, ureagenesis and gluconeogenesis. Int. J. Vitam. Nutr. Res..

[23] Tomizawa M., Shinozaki F., Hasegawa R., Shirai Y., Motoyoshi Y. (2015). Patient characteristics with high or low blood urea nitrogen in upper gastrointestinal bleeding. World J. Gastroenterol..

[24] Calim A. (2025). The role of BUN/creatinine ratio in determining the severity of gastrointestinal bleeding and bleeding localization. North. Clin. Istanb..

[25] Wu K.H., Shih H.A., Hung M.S., Hsiao C.T., Chen Y.C. (2018). The association between blood urea nitrogen to creatinine ratio and mortality in patients with upper gastrointestinal bleeding. Arab J. Gastroenterol..

[26] Afzal A., Gage B.F., Suhong L., Schoen M.W., Korenblat K., Sanfilippo K.M. (2022). Different risks of hemorrhage in patients with elevated international normalized ratio from chronic liver disease versus warfarin therapy, a population-based retrospective cohort study. J. Thromb. Haemost..

[27] Yang X., Ying S., Lv L., Ji Y., Ying J., Ke H. (2025). Association between international normalized ratio-to-albumin ratio and mortality in critically ill patients with gastrointestinal bleeding: A retrospective MIMIC-IV database study. BMC Gastroenterol..

[28] Koseoglu Z., Ozkan O.V., Semerci E., Aslan A., Yetim I., Ucar E., Kuvandik G., Temiz M., Borazan A. (2009). The relationship between mortality and inflammation in patients with gastrointestinal bleeding. J. Int. Med. Res..

[29] Bae S.J., Kim K., Yun S.J., Lee S.H. (2021). Predictive performance of blood urea nitrogen to serum albumin ratio in elderly patients with gastrointestinal bleeding. Am. J. Emerg. Med..

[30] Weng S.C., Shu K.H., Tarng D.C., Tang Y.J., Cheng C.H., Chen C.H., Yu T.M., Chuang Y.W., Huang S.T., Sheu W.H. (2013). In-hospital mortality risk estimation in patients with acute nonvariceal upper gastrointestinal bleeding undergoing hemodialysis: A retrospective cohort study. Ren. Fail..

